# The imaging and pathological features of a mucinous tubular and spindle cell carcinoma of the kidney: a case report

**DOI:** 10.1186/1477-7819-11-34

**Published:** 2013-02-02

**Authors:** Marcela Sampaio Lima, Gyl Eanes Barros-Silva, Renan Augusto Pereira, Roberto Cuan Ravinal, Silvio Tucci Junior, Roberto Silva Costa, Valdair Francisco Muglia

**Affiliations:** 1 Department of Pathology – Faculdade de Medicina Ribeiro Preto, University of Sao Paulo (USP), Av Bandeirantes 3900, 14110-000, Ribeirao Preto, State of Sao Paulo, Brazil; 2 Department of Surgery – Urology Division -Faculdade de Medicina Ribeiro Preto, University of Sao Paulo (USP), Ribeirao Preto, Brazil; 3 Department of Internal Medicine – Imaging Division – Faculdade de Medicina Ribeiro Preto, University of Sao Paulo (USP), Ribeirao Preto, Brazil

**Keywords:** Kidney neoplasm, Mucinous tubular and spindle cell carcinoma, Renal cancer

## Abstract

A mucinous tubular and spindle cell carcinoma (MTSCC) is a rare and recently described kidney neoplasm with distal nephron differentiation. It can affect patients of all ages and is more prevalent among women. In this case report, we present a 50-year-old woman who had a renal mass, which was accidently discovered during an investigation for chronic anemia. The final diagnosis of MTSCC was made after the lesion was removed and a pathology work-up was performed. The clinical, pathological and imaging findings of this rare neoplasm are described in this report.

## Background

Mucinous tubular and spindle cell carcinoma (MTSCC) of the kidney is a rare renal cancer, which was first described in 1998 [1]. It was previously designated under the category of unclassified renal cell carcinoma (RCC) [2] in the World Health Organization (WHO) classification of renal neoplasms. In 2004, it was incorporated as a new entity: a variant of RCC [3,4].

The tumor is considered to be a low-grade carcinoma with a favorable prognosis. Its origin has been debated and some pathologists believe that it is derived from the epithelial cells of the loop of Henle, whereas others have credited its origin to the cells of the collecting duct [3].

There are few reports of MTSCC in medical literature and only two focus on the imaging features of the tumor [5,6]. The objective of this report is to describe the clinical, pathological and imaging findings of a case of renal MTSCC diagnosed at our institution.

## Case presentation

A 50-year-old Caucasianwoman was referred for ultrasound (US) examination during the course of an investigation for hypochromic/microcytic anemia, which was refractory to treatment for 4 years. The patient had no urinary complaints, hypertension or diabetes, and had no history of smoking. The pre-operative serum creatinine and hemoglobin levels were 0.8 mg/dl and 9.1 g/dl (hematocrit 30%), respectively.

**Figure 1:**
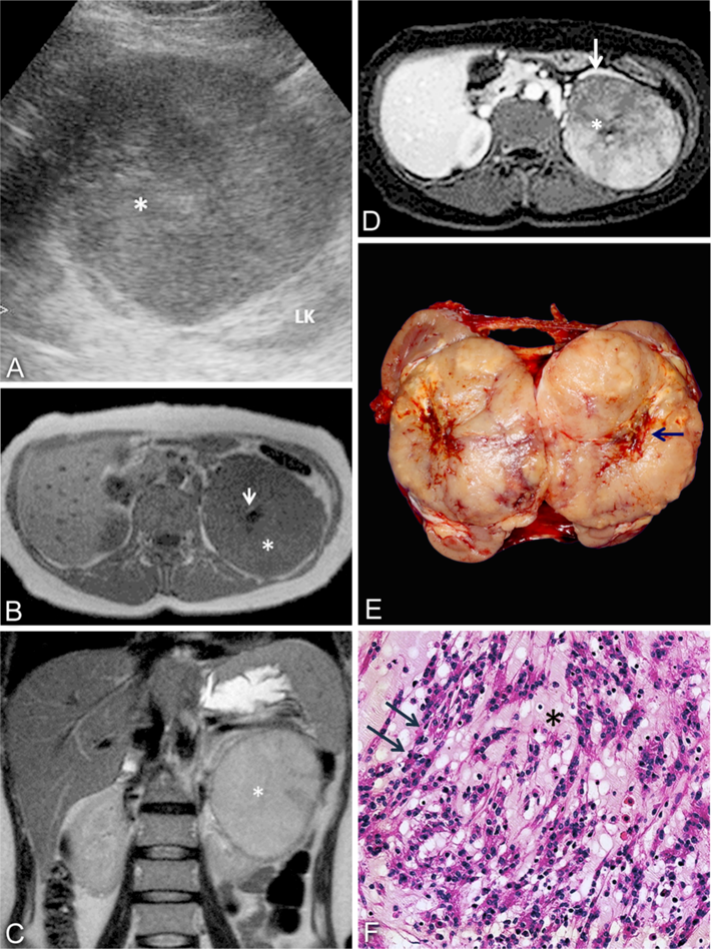
**Imaging findings.** (**A**) Ultrasound image showing a heterogeneous mass (*) in the upper pole of the left kidney (LK)**.** (**B**) Axial T1-weighted and (**C**) coronal T2-weighted imaging demonstrating a mass in the left kidney (*), with homogeneous low T1 and a high signal in T2. A central scar (arrow) is seen on axial T1. (**D**) Axial T1, postcontrast, showing a hypovascular lesion compared to renal cortex (white arrow) with a central scar (*). (**E**) Gross findings: well-circumscribed tumor with a central scar (arrow). (**F**) Elongated tubules and cord arrangements (arrows) embedded in a mucinous stroma (*). LK, left kidney.

Abdominal ultrasonography revealed a round, solid, circumscribed mass, located in the upper pole of the left kidney. The mass was predominantly hypoechoic with echogenic areas in the central portion of the lesion (Figure 1A) and measured 9.5 × 9.0 × 8.0 cm. In view of the uncertain diagnosis, magnetic resonance (MR) was requested. The MR scan confirmed the presence of a circumscribed lesion in the left kidney, with a homogeneous low signal on T1-weighted imaging and an intermediate to high signal on T2-weighted imaging. After intravenous (IV) injection of the contrast medium, there was a diffuse enhancement and most of the lesion was hypovascular compared to the adjacent cortex. On T1-weighted imaging, pre- and postcontrast, a central scar could be defined. There were no signs of vascular, adrenal or perinephric fat invasion. Based on MR findings, a less aggressive and less common histologic variant of RCC, such as chromophobe or papillary lesion, was suspected (Figures 1B, C and D).

The patient underwent a left-sided total nephrectomy and adrenalectomy. The macroscopic examination revealed a single, solid, circumscribed mass, which was primarily white but with yellowish and hemorrhagic areas, and a visible central scar. The longest axis measured about 10.5 cm (Figure 1E).

Histology demonstrated a partially encapsulated tumor composed of tubular and cord arrangements embedded in pale mucinous stroma. The cells were small, cuboidal, with eosinophilic or sometimes vacuolated cytoplasm, and low-grade nuclear features. There were areas with many spindle cells and few mucins. Additionally, foci of epithelioid areas with closely packed round cells without mucin interposed were identified. The central scar showed fibrous connective tissue with lymphoplasmacytic infiltrate. There was no necrosis, vascular or capsular invasion and a sarcomatous component was not defined in the tumor (Figure 1F). No nodal involvement was detected. The Fuhrman nuclear grade was 1 and final pathological staging was pT2b pN0.

Immunohistochemical analysis demonstrated diffuse positivity in the epithelioid and spindle cell areas for cytokeratin (CK) AE1/AE3, 7, 19 and vimentin, and was negative for CD10 and CD15. The immunostaining for epithelial membrane antigen (EMA) was focally positive. Immunostaining for neuron-specific enolase (NSE), chromogranin, synaptophysin, sarcomeric actin and desmin did not show neuroendocrine or muscle differentiation (Table 1).

**Table 1:** Immunohistochemical panel for MTSCC

**Antibodies**	**Results**
AE1/AE3	Positive, diffuse
EMA	Positive, focal
CK7	Positive, diffuse
CK19	Positive, diffuse
Vimentin	Positive, diffuse
CD10, CD15, NSE, synaptophysin, chromogranin, desmin, sarcomeric actin	Negative

CK, cytokeratin; EMA, epithelial membrane antigen; MTSCC, mucinous tubular and spindle cell carcinoma; NSE, neuron-specific enolase.

The patient was followed up for 24 months, with no evidence of local or systemic recurrence, and recovered from anemia.

## Discussion

MTSCC is a rare kidney neoplasm, which predominantly presents in adult women (4:1). The age at presentation varies from 17 to 82 years (mean 53 years) [4,7]. The majority of these tumors are accidentally discovered during abdominal imaging studies [3] due to other indications. Occasionally, when lesions are large, they may present with flank pain or hematuria, causing anemia, as in this case [4]. An association with nephrolithiasis has been described by some authors [8] and was also seen in this case. Complete surgical excision appears to be an adequate treatment and only a few cases have demonstrated recurrence, regional adenopathy or distant metastases, mainly in patients with MTSCC exhibiting components with true sarcomatoid changes [3,6,8-10].

The size of MTSCC is variable. It ranges from less than 1.0 cm in diameter to more than 18.0 cm, with most tumors measuring 2.0 to 4.0 cm in the longest axis [9]. The histological features of the neoplasm are characterized by elongated tubules and cord arrangements, which are separated by variable amounts of mucinous stroma. The parallel tubular arrays often have a spindle cell configuration, sometimes resembling a mesenchymal neoplasm. Cells are small with a cuboid or oval shape and exhibit low-grade nuclear characteristics. Areas of necrosis, solid tubular growth, foam cell deposits and chronic inflammation may be seen in the tumor [4,9].

In the absence of typical morphologic features, a nonclassic MTSCC pattern may be characterized by areas of papillary changes, neuroendocrine differentiation and sarcomatoid changes in the lesion. Immunohistochemistry may be valuable in limiting the potential differential diagnoses, as in this case. MTSCC is generally positive for low molecular weight cytokeratins (CK7, CK19). The epithelial membrane antigen is usually present and vimentin is occasionally detected [4].

Imaging descriptions of MTSCCs are very rare. In Table 2, we present the published MR imaging studies of MTSCCs, including our case. The combination of signal intensity on T1 and T2-weighted imaging with the pattern of enhancement described here is unlikely for the most common variant of RCC, clear cell carcinoma. These tumors usually have areas of high signal on T1-weighted imaging, due to necrosis and hemorrhage, and exhibit a classic hypervascularization after an IV injection of the contrast medium. However, papillary tumors may show a discrete low signal on T2-weighted imaging and a hypovascular pattern after IV contrast medium. The sonographic appearances of these lesions are non-specific.

**Table 2:** Imaging findings of MTSCC described in literature

	**Signal on T2-weighted imaging**	**Morphology**	**Pattern of enhancement**
Noon *et al*. 2010 [4]	Intermediate to high, homogeneous	Circumscribed 7.0 cm	Diffuse, hypovascular
Makni *et al*. 2011 [5]	High, heterogeneous	Circumscribed 18.0 cm	Centripetal enhancement and a central scar
Blinded, 2013	Intermediate to high, homogeneous	Circumscribed 9.5 cm	Diffuse, hypovascular and a central scar

## Conclusion

In conclusion, there are still no specific imaging criteria for the diagnosis of MTSCC, which can only be confirmed by tissue sampling. The imaging features may resemble other variants of RCC, such as chromophobe or papillary types, which have a less favorable prognosis. However, a renal MTSCC should be suspected when a large, circumscribed, poorly enhancing lesion with low to intermediate signal on T2-weighted imaging is discovered, especially if in association with renal lithiasis [3,4].

## Consent

Written informed consent was obtained from the patient for publication of this case report and accompanying images. A copy of the written consent is available for review by the Editor-in-Chief of this journal.

## Abbreviations

CK: Cytokeratin; EMA: Epithelial membrane antigen; IV: Intravenous; MR: Magnetic resonance; MTSCC: Mucinous tubular and spindle cell carcinoma; NSE: Neuron-specific enolase; RCC: Renal cell carcinoma; US: Ultrasound; WHO: World Health Organization.

## Competing interests

The authors declare that they have no competing interests.

## Authors’ contributions

GEBS and VFM act as guarantors of integrity of the entire study. GEBS, RSC and VFM provided study concepts and design. MSL, GEBS, VFM and RCR provided literature research. MSL, RAP and ST performed the clinical studies. MSL, RAP, RSC and RCR prepared the manuscript. GEBS and VFM edited the manuscript. ST provided photographic documentation. All authors read and approved the final manuscript.
